# Acculturation and gestational weight gain in a predominantly puerto rican population

**DOI:** 10.1186/1471-2393-12-133

**Published:** 2012-11-21

**Authors:** Alison Tovar, Lisa Chasan-Taber, Odilia I Bermudez, Raymond R Hyatt, Aviva Must

**Affiliations:** 1 Department of Nutrition and Food Science, University of Rhode Island, 112 Ranger Hall, University of Rhode Island, Kingston, RI 02881, USA; 2 Division of Biostatistics & Epidemiology, School of Public Health & Health Sciences, University of Massachusetts, 405 Arnold House, 715 North Pleasant Street, Amherst, MA, 01003-9304, USA; 3 Department of Public Health and Community Medicine, Tufts University, 136 Harrison Avenue, Boston, MA, 02111, USA

**Keywords:** Gestational weight gain, Acculturation, Pregnancy, Hispanic

## Abstract

**Background:**

Identifying risk factors that affect excess weight gain during pregnancy is critical, especially among women who are at a higher risk for obesity. The goal of this study was to determine if acculturation, a possible risk factor, was associated with gestational weight gain in a predominantly Puerto Rican population.

**Methods:**

We utilized data from Proyecto Buena Salud, a prospective cohort study of Hispanic women in Western Massachusetts, United States. Height, weight and gestational age were abstracted from medical records among participants with full-term pregnancies (n=952). Gestational weight gain was calculated as the difference between delivery and prepregnancy weight. Acculturation (measured via a psychological acculturation scale, generation in the US, place of birth and spoken language preference) was assessed in early pregnancy.

**Results:**

Adjusting for age, parity, perceived stress, gestational age, and prepregnancy weight, women who had at least one parent born in Puerto Rico/Dominican Republic (PR/DR) and both grandparents born in PR/DR had a significantly higher mean total gestational weight gain (0.9 kg for at least one parent born in PR/DR and 2.2kg for grandparents born in PR/DR) and rate of weight gain (0.03 kg/wk for at least one parent born in PR/DR and 0.06 kg/wk for grandparents born in PR/DR) vs. women who were of PR/DR born. Similarly, women born in the US had significantly higher mean total gestational weight gain (1.0 kg) and rate of weight gain (0.03 kg/wk) vs. women who were PR/ DR born. Spoken language preference and psychological acculturation were not significantly associated with total or rate of pregnancy weight gain.

**Conclusion:**

We found that psychological acculturation was not associated with gestational weight gain while place of birth and higher generation in the US were significantly associated with higher gestational weight gain. We interpret these findings to suggest the potential importance of the US “obesogenic” environment in influencing unhealthy pregnancy weight gains over specific aspects of psychological acculturation.

## Background

Pregnancy has been proposed as a critical period for the development of overweight and obesity in mothers, with ramifications for both the mother and the infant [1-6]. Compared to previous decades, women in America of childbearing age currently enter pregnancy at higher weights [7] and are more likely to gain excess weight during pregnancy [3,8,9]. Excess gestational weight gain, in turn, has been associated with pregnancy complications, including cesarean delivery, large-for-gestational-age infants, and postpartum weight retention [10]. Ethnic minorities such as Hispanics are disproportionately affected by overweight and obesity [11-13] and are more likely to begin their pregnancies overweight or obese [12,14,15] with almost half beginning pregnancy in these categories [16-18]. In addition, the number of Hispanic women with both elevated BMI and excessive gestational weight gain has been increasing over time [8,9]. Therefore, identifying risk factors that affect excess weight gain during pregnancy among Hispanic women is critical.

One potential risk factor for excess gestational weight gain in Hispanic women is acculturation. Acculturation, defined as a process that entails contact between two cultural groups, which results in numerous cultural changes in both parties [19], has been found to be an important predictor of health-risk among women generally [20]. For example, a cross-sectional study of 174 low-income Puerto Rican women found that high levels of acculturation (measured by years of residence in the US and language preference) were associated with changes in food habits, such as higher consumption of soda, fruit juice and snack food [21]. Regardless of the country of origin, upon arrival, Hispanic immigrants are healthier than US-born adults but these health advantages dissipate over time [22]. In particular, studies have shown that overweight and obesity increase with length of stay in the US [23-28]. This rise in weight may be influenced by the “obesogenic” environment of the US, characterized by the availability of energy-dense, palatable, inexpensive foods and limited opportunities for physical activity [23-26,29,30].

According to the 2000 Census, nearly 67 million people of Hispanic origin will be added to the nation’s population between 2000 and 2050. Their numbers are projected to grow from 35.6 million to 102.6 million, an increase of 188 percent [31]. The Hispanic population is heterogeneous with many sub-groups. The largest group in the US is Mexican-American but other groups, such as Central, Caribbean and South Americans are large and growing [31]. Disparities in maternal and infant health are substantial in this population; Hispanic women tend to be more physically inactive and have almost double the prevalence of prepregnancy diabetes, as compared to non-Hispanic white women. Rates of breastfeeding are also low [32]. The elimination of health disparities is one of the major goals of Healthy People 2020 and further understanding acculturation may help in doing so. According to the latest Institute of Medicine (IOM) report that reviewed gestational weight gain guidelines, only a few studies had examined the effects of acculturation on gestational weight gain and therefore the IOM has called for further research in this area [33].

To our knowledge few studies have examined the impact of acculturation on gestational weight gain in Hispanic women. In addition to measures of acculturation based on demographic factors such as place of birth, multidimensional scales can integrate the directionality of several dimensions of an individual’s acculturative experience. Therefore, the purpose of this study was to determine if acculturation, as measured by a psychological acculturation scale, as well as place of birth, generation, and spoken language preference, were associated with weight gain during pregnancy in a predominantly Puerto Rican population. We hypothesized that higher levels of all acculturation measures would be associated with increased weight gain during pregnancy.

## Methods

### Description of study design

We utilized data from Proyecto Buena Salud, a prospective cohort study of Hispanic women. Recruitment into this study began in January 2006 [34]. The study was based in the public obstetrics and gynecology clinic and midwifery practice at Baystate Medical Center, a large tertiary care facility in Western Massachusetts which serves a large predominately Puerto Rican Hispanic population. Medical nutritional therapy is routinely provided to Baystate Medical Center patients, and includes general dietary counseling, dietary counseling for medical conditions (such as diabetes or pregnancy related diabetes), breastfeeding information, and referrals to the WIC program. Weight goals follow those recommended by the IOM [33]. Bilingual interviewers recruited patients at a prenatal care visit early in pregnancy (up to 20 weeks gestation), informed them of the aims and procedures of the study, and obtained written informed consent (in English or Spanish) as approved by the Institutional Review Boards of the University of Massachusetts-Amherst and Baystate Health. Interviews were conducted in Spanish or English (based on patient preference) in order to eliminate potential language barriers. At the time of recruitment, interviewers collected information on socio-demographic factors, prepregnancy BMI, physical activity, psychosocial stress, cigarette smoking, and acculturation.

After delivery, medical records were abstracted for medical and obstetric history, clinical characteristics of the current pregnancy and birth outcomes. Eligibility was restricted to women of Puerto Rican or Dominican Republic heritage (Caribbean Islanders). Women who: 1) were themselves born in the Caribbean Islands, or 2) had a parent born in the Caribbean Islands, or 3) had at least 2 grandparents born in the Caribbean Islands were included. Exclusion criteria were: 1) current use of medications that could influence glucose tolerance, 2) multiple gestation, 3) history of diagnosis of diabetes prior to pregnancy, hypertension, heart disease or chronic renal disease, and 4) age <16 years or > 40 years. We restricted the current analysis to those participants with a full-term, live birth pregnancy who delivered at Baystate Medical Center between 2006 and 2011 (n=1570). We excluded women who experienced a preterm birth (<37 weeks gestation), miscarried or terminated their pregnancy (n=86), or were missing data on acculturation (n=247) or missing prepregnancy weight or weight at the time of delivery (n=300).

### Assessment of gestational weight gain

Gestational weight gain was calculated as the difference between maternal weight at delivery and prepregnancy weight. Women self-reported their prepregnancy weight at their first prenatal care visit at which time it was recorded in their medical record. After delivery, the prepregnancy weight was abstracted from the medical record report. We excluded 15 women whose reported pregravid weights differed by more than 9 kg from their weight measured at a prenatal care visit prior to 12 weeks gestation [35]. In addition, if prepregnancy weight was missing from the record (n=176), weight at the time of first prenatal visit before 13 weeks gestation was used. A weight gain value of 50 kg of weight loss was excluded as an outlier (n=1) as it exceeded four times the SD. This resulted in a final sample size of 952 women.

Rate of weight gain was calculated as the difference between maternal weight at delivery and prepregnancy weight, divided by gestational age at delivery in weeks. For each subject, we calculated total weight (kg) and rate of gestational weight gain (kg/w). In addition to classifying gestational weight gain as a continuous variable, we also created a categorical variable to evaluate weight gain according to the new IOM gestational weight gain guidelines [33]: gaining less than recommended, gaining the recommended amount, or gaining more than recommended. These guidelines vary by prepregnancy BMI such that “underweight” women (BMI < 18.5 kg/m^2^) are advised to gain 13–18 kg, “normal weight” women (BMI 18.5–24.9 kg/m^2^) 11–16 kg, and “overweight women” (BMI 25–29.0 kg/m^2^) 7–11 kg. For women with a prepregnancy BMI ≥30.0 kg/m^2^, a recommended target weight gain between 5 and 9 kg is specified by the IOM. Because the data were collected prior to the 2009 IOM recommendations, this study was not designed to be a practical assessment of compliance with these guidelines.

### Assessment of acculturation

Acculturation was assessed via several measures. We measured psychological acculturation using the Psychological Acculturation Scale (PAS). The PAS is an acculturation assessment tool with multiple items that pertain to the individual’s sense of psychological attachment or belonging within Anglo-American and Hispanic culture, as well as an individual’s psychological negotiation between these two cultural entities [36]. Reliability and validity of the PAS has been demonstrated in a sample of bilingual Puerto Rican respondents [36]. The PAS encompasses four dimensions (cultural loyalty, solidarity, comprehension, and identification) shown to reflect psychological responses to cultural exposure. It is expressed as the mean of 10 items, which are rated from 1 (only Hispanic/Latino orientation) to 5 (only Anglo-American orientation), with a bicultural orientation at the midpoint. Psychological acculturation was assessed as a continuous variable and as a categorical variable by tertiles. In addition, generation, place of birth (US vs. Puerto-Rico [PR]/ Dominican Republic [DR]), and spoken language preference (English vs. Spanish) were used as measures of acculturation. The question on language preference was investigator-derived. Specifically, women were asked if they preferred to speak and read, respectively, in English, Spanish or another language. Generation in the US was derived from questions based on the US Census. That is, women were asked if they self-identified as Hispanic or Latino. Those who responded affirmatively were asked to report their birthplace, their parents’ birthplace, and their grandparents’ birthplace.

### Assessment of covariates

We collected information on known or suspected risk factors for gestational weight gain, including maternal age, education (highest level of education completed), annual household income, parity, prepregnancy BMI, perceived stress, physical activity and cigarette smoking during pregnancy [10]. Perceived stress has been associated both with gestational weight gain [37] and acculturation [38] and was measured using Cohen’s Perceived Stress Scale (PSS-14) which has been previously validated [39]. [40] and includes 14 items designed to assess a person’s sense of control over daily life demands [40]. Physical activity was measured using a modified version of the Pregnancy Physical Activity Questionnaire (PPAQ), a validated semi-quantitative questionnaire that assesses the duration, frequency, and intensity of total physical activity [41].

### Statistical analysis

Data management and analysis were conducted in SAS (version 9.2). We utilized one-way analysis of variance to compare overall means for continuous variables and bivariate analysis using χ^2^ to test for differences in categorical variables. Using multiple linear regression we regressed total and rate of weight gain on acculturation measures. Multinomial logistic regression was used to regress the different levels of IOM recommendations on acculturation. Confounding was assessed by evaluating the change in the acculturation β-coefficients when each covariate was included in the regression model (for PAS tertiles, the top tertile was used to assess change). A 10% or greater change was deemed to indicate confounding was present.

Statistical interaction between prepregnancy BMI and psychological acculturation as well as place of birth and psychological acculturation were evaluated by assessing the statistical significance of cross-product terms in the regression models using the F-test. For covariates with missing values, a missing value category was created and used to maximize the sample size for analysis.

## Results

The 952 women were on average 22.7± 4.9 years old, 46.8% had less than a high school education and 42.8% were nulliparous. The mean level of psychological acculturation as measured by the PAS was 2.4± 0.6 (with a possible range of 1 to 5). A total of 54.4% of women were born in the US, 75.9% preferred to speak English, and 5.2% were 3^rd^ generation in the US (Table 1). Almost half of women were overweight or obese prior to pregnancy (42.1%) and mean physical activity levels were 36.1 (SD=24.6) MET-hrs/week (representing approximately 6 hours of moderate-intensity activity per week). A total of 12.9% of women smoked during pregnancy and mean stress was 26.0 (possible range 0 to 56). Participant age, education level, parity, BMI, smoking during pregnancy, perceived stress score, and physical activity level did not differ across those with low to high acculturation according to PAS tertiles. As expected, women with high levels of psychological acculturation were more likely to be born in the US (72.3%) as compared to women with low levels of psychological acculturation (36.5%, p<0.001). Similarly, women with high levels of psychological acculturation were more likely to prefer to speak English (95%) as compared to women with low levels of psychological acculturation (52.2%, p<0.0001). Finally, women with high levels of psychological acculturation were more likely to be third generation (8.2%) as compared to women with low levels of psychological acculturation (2.4%, p<0.0001).

**Table 1:** Characteristics of the study population according to psychological acculturation status;proyecto buena balud, Western Massachusetts, 2006-2011

	**Total population**		**Low acculturation**		**Mid acculturation**		**High acculturation**	
	**(n=952)**		**PAS <2.2**		**PAS 2.2-2.8**		**PAS ≥2.8**	
	**N**	**%**	**(n=305)**		**(n=325)**		**(n=322)**	
			**N**	**%**	**N**	**%**	**N**	**%**
**Socio-demographic Variables**								
Age (mean, SD)	22.7	4.9	22.9	5.2	22.3	4.6	22.8	4.9
Education								
Less than high school	445	46.8	149	48.9	155	47.8	141	43.9
High school, trade/technical school	322	33.9	108	35.4	107	33.0	107	33.3
Some college/college graduate/graduate	183	19.3	48	15.7	62	19.4	73	22.7
**Acculturation Variables**								
Psychological Acculturation Scale (PAS) (mean, SD)	2.4	0.6	1.7	0.3	2.5	0.2	3.1	0.3
Birthplace**								
PR/DR born	427	45.6	186.0	63.5	147	46.8	88	27.7
U.S. born	509	54.4	107.0	36.5	167	53.2	230	72.3
Spoken language preference**								
Spanish	214	23.6	139	46.8	60	20.0	15	5.0
English	687	75.9	155	52.2	244	80.0	288	95.0
Generation^**^								
Born in PR/DR	421	45.7	186	63.5	147	47.0	88	27.9
At least one parent born in PR/DR	452	49.1	100	34.1	150	48.1	202	63.9
Grandparents born in PR/DR	48	5.2	7	2.4	15.0	4.8	26	8.2
**Medical Variables**								
Parity								
0	407	42.8	124	40.8	144	44.3	139	43.3
1	301	31.7	105	34.5	104	32.0	92	28.7
≥2	242	25.5	75	24.7	77	23.7	90	28.0
BMI Categories (kg/m^2^)								
Underweight <18.5	66	7.0	14	4.6	27	8.3	25	7.8
Normal weight 18.5-25	484	51.0	150	49.3	164	50.5	170	53.0
Overweight 25-30	195	20.5	73	24.0	62	19.1	60	18.7
Obese ≥ 30	205	21.6	67	22.0	72	22.2	66	20.6
**Behavioral Variables**								
Smoked during pregnancy	106	12.9	33	12.2	37	13.0	36	13.5
Perceived Stress Scale (mean, SD)	26.0	7.0	26.8	6.9	25.5	6.7	25.6	7.6
Total Physical activity (MET [Metabolic Equivalents]/wk.)	36.1	24.6	36.4	28.6	34.5	24.0	37.3	20.6

Sample sizes vary slightly due to missing data.

*p<0.01,**p<0.0001.

P-values generated from Chi-square tests.

The mean total weight gain in this sample was 14.0 kg (SD=6.8) with a mean weekly weight gain of 0.36 kg (SD=0.2) per week (Table 2). Although women with high levels of acculturation as measured by the PAS had higher mean total weight gain compared to women with low levels of acculturation (14.4 vs. 14.3 kg) this difference was not statistically significant. In addition, we observed no statistically significant correlations between PAS score and total gestational weight gain (r=0.01, p=0.7) or rate of weight gain (r=0.01, p=0.8). However, women born in the US had a greater mean total weight gain (14.6 kg) and higher rate of weight gain (0.37 kg/wk) compared to women born in PR/DR (13.3 kg and 0.34 kg/wk, p<0.01 and p=0.02, respectively). Similarly, women of grandparents born in PR/DR had greater mean total weight gain (16.3 kg) and rate of weight gain (0.41 kg/wk) compared to PR/DR born women (13.3 kg and 0.34 kg/wk, p<0.01 and p=0.01, respectively). Mean total gestational weight gain and mean rate of weight gain did not differ significantly according to language preference.

**Table 2:** Acculturation variables according to gestational weight gain proyecto buena salud, Western Massachusetts, 2006-2011

	**Gestational weight gain**				**Meeting IOM guidelines†**						
	**Total weight gain (n=952) (kg)**		**Rate of weight gain (n=947) (kg/week)**		**Above**		**Within**		**Below**		p value*
	**mean**	**SD**	**Mean**	**SD**	**N**	**%**	**N**	**%**	**N**	**%**	
Total Sample	14	6.8	0.36	0.2	440	46.3	295	31.1	215	22.6	
Psychological acculturation scale											
Low acculturation	14.3	6.5	0.36	0.2	150	49.3	93	30.6	61	20.1	
Mid acculturation	13.4	7.1	0.34	0.2	139	42.8	101	31.1	85	26.2	
High acculturation	14.4	6.7	0.36	0.2	151	47.0	101	31.5	69	21.5	
P-value*	0.13		0.14								0.4
Generation											
Born in PR/DR	13.3	6.7	0.34	0.2	178	42.5	140	33.4	101	24.1	
At least one parent born in PR/DR	14.4	6.9	0.36	0.2	219	48.5	130	28.8	103	22.8	
Grandparents born in PR/DR	16.3	5.7	0.41	0.1	30	62.5	13	27.1	5	10.4	
P-value*	<0.01		0.01								0.04
Birthplace											
PR/DR born	13.3	6.7	0.34	0.2	178	42.5	140	33.4	101	24.1	
US born	14.6	16.9	0.37	0.2	250	49.6	144	28.6	110	21.8	
P-value*	<0.01		0.02								0.09
Language preference											
Spanish	13.4	6.4	0.35	0.2	97	46.3	68	31.8	49	22.9	
English	14.1	6.9	0.36	0.2	317	45.3	211	30.8	157	22.9	1.0
P-value*	0.2		0.4								

**†** “underweight"(BMI < 18.5 kg/m2) advised to gain 13–18 kg, “normal weight” (BMI 18.5–24.9 kg/m2).

11–16 kg, “overweight” (BMI 25–29.0 kg/m2) 7–11 kg "obese" BMI ≥30.0 kg/m2, advised to gain 5–9 kg.

Sample sizes vary slightly due to missing data.

*Statistical significance based on ANOVA and Chi-square calculations.

A total of 31.1% of the sample met the IOM guidelines, while 46.3% exceeded these guidelines and 22.6% fell short of meeting the guidelines (Table 2). A higher percentage of women who had grandparents born in PR/DR exceeded the IOM guidelines (62.5%) as compared to women who were PR/DR born (42.5%), p=0.04. There were no statistically significant differences in meeting IOM guidelines according to psychological acculturation as measured by the PAS, birthplace nor spoken language preference (Table 2).

In unadjusted linear regression analysis, increasing acculturation as measured by a one-unit change in PAS score, was associated with a mean increase of 0.12 kg in total weight gain and a 0.003 kg/wk increase in rate of pregnancy weight gain but these increases were not statistically significant (Table 3). Findings were virtually unchanged when adjusting for key risk factors associated with weight gain. Adjusting for age, parity, gestational age, and prepregnancy weight, women born in the US had significantly higher mean total gestational weight gain (1.0 kg) and rate of weight gain (0.03 kg/wk) as compared to women who were PR/DR born. Similarly, adjusting for age, parity, perceived stress, gestational age and prepregnancy weight, women who had at least one parent born in PR/DR and both grandparents born in PR/DR had a significantly higher mean total gestational weight gain (0.9 kg for at least one parent born in PR/DR and 2.2 kg for grandparents born in PR/DR) and rate of weight gain (0.03 kg/wk for at least one parent born in PR/DR and 0.06 kg/wk for grandparents born in PR/DR) as compared to women who were PR/DR born. Spoken language preference was not significantly associated with total or rate of pregnancy weight gain (Table 3).

**Table 3:** Beta coefficients and standard errors from a linear regression model for weight gain;proyecto buena salud, Western Massachusetts, 2006-2011

	**Total weight gain (kg)**						**Rate of weight gain (kg/wk)**					
	**(n=952)**						**(n=947)**					
	**Unadjusted model**			**Adjusted model**			**Unadjusted model**			**Adjusted model**		
	**B**	**SE**	**p value**	**B**	**SE**	**p value**	**B**	**SE**	**p value**	**B**	**SE**	**p value**
Psychological Acculturation Scale												
Continuous acculturation score^1^	0.12	0.3	0.71	0.15	0.3	0.65	0.003	0.009	0.76	0.005	0.009	0.58
Low acculturation^2^	referent			referent			referent			referent		
Mid acculturation	-.09	0.5	0.10	−0.9	0.5	0.10	−0.02	0.01	0.11	−0.02	0.01	0.11
High acculturation	0.1	0.5	0.85	0.07	0.5	0.89	0.002	0.01	0.85	0.003	0.01	0.79
Generation^3^												
Born in PR/DR	referent			referent			referent			referent		
At least one parent born in PR/DR	1.1	0.5	0.02	0.9	0.4	0.04	0.02	0.01	0.05	0.03	0.01	0.03
Grandparents born in PR/DR	3.0	1.0	<0.01	2.2	1.0	0.03	0.07	0.03	<0.01	0.06	0.03	0.02
Birthplace^4^												
PR/DR born	referent			referent			referent			referent		
US Born	1.2	0.4	<0.01	1.0	0.4	0.02	0.02	0.01	0.02	0.03	0.01	0.01
Language preference^5^												
Spanish	referent			referent			referent			referent		
English	0.7	0.5	0.19	0.09	0.5	0.86	0.01	0.01	0.32	0.003	0.01	0.81

Adjusted for age, parity, education, physical activity, gestational age and perceived stress.

Adjusted for age, gestational age and perceived stress.

Adjusted for age, parity, perceived stress, gestational age and prepregnancy weight.

Adjusted for age, parity, gestational age and prepregnancy weight.

Adjusted for age, parity, gestational age and prepregnancy weight.

We then evaluated the association between acculturation and meeting IOM weight gain guidelines (Table 4). In unadjusted and adjusted multinomial logistic regression analysis, psychological acculturation and spoken language preference were not significantly associated with exceeding or not achieving IOM guidelines. Compared to women who were PR/DR born, women who had grandparents born in PR/DR were almost two times more likely to exceed the IOM guidelines (OR=1.9, 95% CI 1.0-3.9) adjusting for age, parity, gestational age and prepregnancy weight, this was of marginal significance (Table 4). Similarly, compared to women who were PR/DR born women who were US born were 1.4 times more likely to exceed the IOM guidelines (OR=1.4, 95% CI 1.0-1.9) adjusting for age, gestational age and parity. Finally, prepregnancy BMI and place of birth did not significantly modify the relationship between psychological acculturation and weight gain.

**Table 4:** Multivariable odds ratios (OR) and 95% confidence intervals (CI) for meeting IOM guidelines using multinomial logistic regression; proyecto buena salud, Western Massachusetts, 2006-2011

	**Meeting IOM guidelines**							
	**Above**				**Below**			
	**OR**	**95% CI**		**p-value**	**OR**	**95% CI**		**p-value**
Psychological Acculturation Scale								
Continuous acculturation score^1^	1.0	0.8	1.3	0.94	1.0	0.8	1.4	0.71
Low acculturation^2^	1.0	referent			1.0	referent		
Mid acculturation	0.8	0.6	1.2	0.27	1.3	0.8	2.0	0.29
High acculturation	1.0	0.7	1.4	0.82	1.0	0.7	1.7	0.79
Generation^3^								
Born in PR/DR	1.0	referent			1.0	referent		
At least one parent born in PR/DR	1.3	0.9	1.8	0.10	1.2	0.8	1.7	0.44
Grandparents born in PR/DR	1.9	1.0	3.9	0.06	0.6	0.2	1.7	0.30
Birthplace^4^								
PR/DR born	1.0	referent			1.0	referent		
US born	1.4	1.0	1.9	0.05	1.1	0.7	1.6	0.53
Language preference^5^								
Spanish	1.0	referent			1.0	referent		
English	1.1	0.7	1.5	0.75	1.1	0.7	1.6	0.79

^1^Model adjusted for age, education, parity, gestational age, and prepregnancy weight.

^2^Model adjusted for age, parity, perceived stress gestational age, and prepregnancy weight.

^3^ Model adjusted for age, parity gestational age, and prepregnancy weight.

^4^ Model adjusted for age gestational age, and parity.

^5^Model adjusted for age, parity, gestational age, and prepregnancy weight.

OR=Odds Ratios;CI=Confidence Intervals.

## Discussion

In this prospective study of Hispanic women, participants who were US born gained on average 1.0 kg more weight over the course of pregnancy and were 1.4 times more likely to exceed the guidelines as compared to PR/DR born women. Weight gain increased with generation in the US. For example, women who had grandparents born in PR/DR gained on average 2.2 kg more over the course of their pregnancy and were approximately two times more likely to exceed IOM pregnancy weight gain guidelines as compared to women who were PR/DR born. Psychological acculturation, as measured by the PAS, as well as spoken language preference were not significantly associated with pregnancy weight gain or exceeding the IOM guidelines.

Our finding that women who were more acculturated, as reflected by their place of birth and generation status, gained more weight is similar to what others have found in studies of pregnant [42] and non-pregnant US populations [22,43,44]. A study of 1,597 Hispanic women who were part of the New Mexico Pregnancy Risk Assessment Monitoring System in 2000, found that compared to residence in a non-border county, residence in a US Mexico border county reduced such risk of excess gestational weight gain (OR=0.75, 95% CI=0.59, 0.97) [45]. Among 773 women of Mexican descent who gave birth in one of three rural California hospitals, Heilemann and colleagues found that 60% of US born women gained over 18 kg compared to 33% of Mexican born women [46]. They also found that the majority of women born in the US exceeded weight gain guidelines while those who were Mexican born did not. Similarly, a 2010 study completed among 259 pregnant Hispanic women in New York City found that language preference was not associated with prenatal weight gain [47], consistent with our findings.

Given the limited number of studies completed in pregnancy, considering studies conducted among non-pregnant women of child bearing age may help inform our findings. For example, in non-pregnant women, using data from the National Interview Survey, Goel found that after adjusting for age, socio-demographic and lifestyle factors, living in the United States for 10–15 years was associated with increases of 0.88 BMI units compared to those who had been living in the US from 0–5 years [22]. Similarly, Fuentes-Afflick found that length of time residing in the US was associated with obesity among Hispanic women of childbearing age (OR=1.08 for each additional year residing in the US, 95%CI 1.02-1.25) [42]. It is worth noting, that most of these studies were conducted among Mexican-American women, but our findings in this predominantly Puerto Rican population are consistent with these populations in spite of the fact that Puerto Rico is considered a US territory, thus reducing barriers to travel to and from the US mainland.

One interpretation of our finding that place of birth and generation were significantly related to both gaining more weight during pregnancy and exceeding the IOM pregnancy weight gain guidelines is that the environment plays a greater role in influencing unhealthy pregnancy weight gains than specific aspects of psychological acculturation, such as feeling proud or sharing beliefs and values of a certain culture. Exposure to and residence within this US “obesogenic” environment, characterized by the readily available energy dense, palatable, inexpensive foods and limited opportunities for physical activity, likely contributes to weight gain [23-26,29,30,48-54]. Opportunities for nutrition education and anticipatory guidance regarding the “obesogenic” environment and how to navigate it, for ethnic minority women who have been exposed for longer periods of time in particular, could be made available through existing prenatal care services such as WIC and other nutrition education venues.

Several limitations of our study are worth noting. First, like most studies of gestational weight gain, prepregnancy weight was self-reported, and, as such, may have been misreported. However, studies have shown that recalled prepregnancy weight correlates well with measured weights [55,56]. To minimize this error however, first clinic visit weights were reviewed against prepregnancy weights for biologic plausibility as has previously been done [35]. It is worth noting that, women with missing prepregnancy weights were more likely to be first time mothers (29% vs. 43%, p< 0.01) or to have had 2 or more pregnancies (47% vs.26%); more likely to be born in PR (59% vs. 46%, p=0.05) and prefer to speak Spanish (40% vs. 24%, p<0.01), as compared to those not missing prepregnancy weights, which may have biased our results. Given that our findings suggest that PR/DR born gain less weight during pregnancy it is possible that our results are an underestimation of the true effect. Second, Hispanic women in the current study were, by design, limited to those with ancestral roots in Puerto Rico or the Dominican Republic, reflecting the high percentage of these groups in New England. The generalizability of these results to other Hispanic women (e.g., Mexican, Central American) is thus unknown. Third, information on the nature of weight gain recommendations women received during their pregnancy was not captured. From 54 pregnant women from the study population, approximately 24% report that they received weight gain recommendations from their doctor. These findings are similar to results from focus groups where women did not recall receiving a weight gain recommendation, especially if they were overweight or obese [57]. Fourth, because only 5% of women had grandparents born in PR/DR, these results should be interpreted cautiously. Fifth, although we hypothesized that there would be more variability in the PAS measure, its focus on psychological measures of acculturation may reduce its ability to detecting true differences in acculturation which, in this population, may be expressed more via behavioral changes in, for example, diet and physical activity and risk-taking behaviors. Finally, although information on place of birth and generation in the US was available for our participants, length of time in the US mainland and frequency of travel to Puerto Rico and the Dominican Republic was not assessed, and may have been meaningful measures. It is increasingly recognized that acculturation is a complicated and individual process [58]. Thus, we may not have fully captured the aspects of acculturation that affect pregnancy weight gain.

## Conclusion

In summary, in this cohort of predominately Puerto Rican women, women who were US born and those with increasing generation in the US had higher mean gestational weight gain and were more likely to exceed the IOM guidelines for weight gain in pregnancy compared to PR/DR born women. Psychological acculturation, as measured by PAS, along with spoken language preference for speaking were not significantly associated with gestational weight gain. Future studies should confirm these findings and continue to explore the complex nature of acculturation while considering different modifiable risk factors that may influence this process, such as diet and physical activity. Given that Hispanic women are at increased risk for developing obesity, creating culturally appropriate interventions for Hispanic women during pregnancy may aid in the prevention of this critical public health problem.

## Competing interests

The authors declare that they have no competing interests.

## Authors’ contributions

All the authors contributed to the various stages of this study. AT contributed to the study design, performed all of the statistical analysis, and drafted the manuscript. OB and RH participated in the design of the study and revised manuscript. AM and LCT conceived of the initial idea of the study, contributed to design of the study, revised the manuscript and contributed especially to the intellectual content. All the authors read and commented on the drafts and approved of the final version for submission.

## Pre-publication history

The pre-publication history for this paper can be accessed here:

http://www.biomedcentral.com/1471-2393/12/133/prepub
